# Duloxetine Inhibits Microglial P2X4 Receptor Function and Alleviates Neuropathic Pain after Peripheral Nerve Injury

**DOI:** 10.1371/journal.pone.0165189

**Published:** 2016-10-21

**Authors:** Tomohiro Yamashita, Shota Yamamoto, Jiaming Zhang, Miho Kometani, Daisuke Tomiyama, Keita Kohno, Hidetoshi Tozaki-Saitoh, Kazuhide Inoue, Makoto Tsuda

**Affiliations:** 1 Department of Molecular and System Pharmacology, Graduate School of Pharmaceutical Sciences, Kyushu University, Fukuoka, 812-8582, Japan; 2 Department of Life Innovation, Graduate School of Pharmaceutical Sciences, Kyushu University, 3-1-1 Maidashi, Higashi-ku, Fukuoka, 812-8582, Japan; Tokyo Metropolitan Institute of Medical Science, JAPAN

## Abstract

P2X4 receptors (P2X4R) are a family of ATP-gated non-selective cation channels. We previously demonstrated that activation of P2X4R in spinal microglia is crucial for neuropathic pain, a highly debilitating chronic pain condition, suggesting that P2X4R is a potential therapeutic target for treating neuropathic pain. Thus, the identification of a compound that has a potent inhibitory effect on P2X4R is an important clinical challenge. In the present study, we screened a chemical library of clinically approved drugs and show for the first time that duloxetine, a serotonin and noradrenaline reuptake inhibitor, has an inhibitory effect on rodent and human P2X4R. In primary cultured microglial cells, duloxetine also inhibited P2X4R-, but not P2X7R-, mediated responses. Moreover, intrathecal administration of duloxetine in a model of neuropathic pain produced a reversal of nerve injury-induced mechanical allodynia, a cardinal symptom of neuropathic pain. In rats that were pretreated with a serotonin-depleting agent and a noradrenaline neurotoxin, the antiallodynic effect of duloxetine was reduced, but still remained. Based on these results, we suggest that, in addition to duloxetine’s primary inhibitory action on serotonin and noradrenaline transporters, an inhibitory effect on P2X4R may be involved at least in part in an antiallodynic effect of intrathecal duloxetine in a model of neuropathic pain.

## Introduction

Neuropathic pain is a source of intractable chronic pain in conditions such as cancer, fibromyalgia, diabetic neuropathy and postherpetic neuralgia. A prominent symptom is abnormal pain hypersensitivity evoked by normally innocuous stimuli, known as mechanical allodynia. Analgesics such as pregabalin and opioids are used for treating neuropathic pain, however it has been estimated that only one in four patients experiences over 50% pain relief [1]. Furthermore, the clinical use of these drugs is often limited by side effects such as dizziness, obstipation and nausea [2]. Thus, there is a need to explore and develop analgesics with therapeutic potential for neuropathic pain.

Microglia are the resident immune cells in the central nervous system [3]. Following peripheral nerve injury (PNI), microglia critically contribute to the pathogenesis of neuropathic pain [4,5,6,7,8]. We have previously shown that the purinergic receptor P2X4 (P2X4R), a subtype of ATP-gated non-selective cation channels, is highly upregulated in spinal microglia after PNI, and blocking the function of the P2X4R produces a reversal of mechanical allodynia [4]. Furthermore, disruption of the P2X4R gene abolishes mechanical allodynia induced by PNI [9,10]. Interfering with microglial P2X4R upregulation by inhibiting interferon regulatory factor 8 and 5 also suppresses PNI-induced allodynia [6,7]. These findings indicate that activation of P2X4R in spinal microglia plays a pivotal role in the pathogenesis of neuropathic pain and suggest that P2X4R blockers are a promising therapeutic approach for neuropathic pain [11,12].

The aim of this study was to identify clinically approved drugs that inhibit P2X4R. In a recent small scale screening of clinically approved antidepressants and anticonvulsants, we showed that paroxetine, a selective serotonin (5-HT) reuptake inhibitor (SSRI), has an inhibitory effect on P2X4R and produces an antiallodynic effect in an animal model of neuropathic pain [13]. In this study, we performed a large scale screening of a chemical library (1979 clinically approved compounds) to identify a drug with a more potent inhibitory effect on P2X4R than paroxetine, and tested the effects in an animal model of neuropathic pain.

We found that duloxetine, a 5-HT and noradrenaline (NA) reuptake inhibitor (SNRI), has an inhibitory effect on the function of microglial P2X4R. Furthermore, intrathecal duloxetine attenuated PNI-induced mechanical allodynia, an effect that remained even in rats that had been pretreated with the 5-HT depleting agent parachlorophenylalanine (PCPA) and the NA neurotoxin N-(2-chloroethyl)-N-ethyl-2-bromobenzylamine (DSP-4). Therefore, it is possible that, in addition to duloxetine’s inhibition of 5-HT and NA transporters, inhibition of P2X4R may be involved in its antiallodynic effect in a model of neuropathic pain.

## Materials and Methods

### Reagents

Reagents were obtained from the following sources: adenosine 5’-triphosphate disodium salt (ATP), 2’(3’)-O-(4-Benzoylbenzoyl)adenosine 5’-triphosphate triethylammonium salt (BzATP), adenosine 5'-diphosphate sodium salt (ADP), amitriptyline hydrochloride, bupropion hydrochloride, clomipramine hydrobromide, maprotiline hydrochloride, mirtazapine, ivermectin, 4-Chloro-DL-phenylalanine methyl ester hydrochloride (PCPA), and N-(2-chloroethyl)-N-ethyl-2-bromobenzylamine (DSP-4) from Sigma-Aldrich (St. Louis, MO, USA); Fura-2-AM from Dojindo (Kumamoto, Japan); duloxetine hydrochloride and paroxetine hydrochloride from Toronto Research Chemicals (Toronto, Canada); pyridoxal phosphate-6-azophenyl-2’,4’-disulphonic acid (PPADS) tetrasodium salt from Tocris Bioscience (Bristol, UK). The approved drugs library of 1979 compounds were provided in the form of 96-well plate format by Drug Discovery Initiative, DDI, The University of Tokyo (Tokyo, Japan).

### Cell cultures

1321N1 human astrocytoma cells stably expressing rat (r) P2X4R, rP2X7R and human (h) P2X4R were used. Cells were maintained in low-glucose DMEM supplemented with 10% heat-inactivated FBS, penicillin and streptomycin in a humidified atmosphere of 5% CO_2_ at 37°C. Primary cultured microglia were prepared according to a previously described method [14,15]. In brief, the mixed glial culture was prepared from brain of neonatal Wistar rats (Kyudo, Saga, Japan) and maintained for 9–24 days in DMEM with 10% heat-inactivated FBS. The mouse microglial cell line C8-B4 cells were maintained in high-glucose DMEM with 4 mM GlutaMax supplemented with 10% heat-inactivated FBS, penicillin and streptomycin in a humidified atmosphere of 5% CO_2_ at 37°C.

### Animals

Male Wistar rats (250–270 g; Japan SLC, Shizuoka, Japan) were housed in individual cages. Neonatal Wistar rats (Kyudo, Saga, Japan) were used for experiments in primary cultured microglia. The animals were housed in groups of four per cage with wooden chip on the floor during habituation, and then in individual cages after intrathecal catheterization under controlled temperature (22 ± 1°C) and humidity (55 ± 10%). The room was lighted from 8:00 AM to 8:00 PM. Food and water were freely available. Standard food (Clea Japan, Tokyo, Japan) and tap water were freely available. The physical conditions of the animals were carefully monitored every other day during the animal experiments. In this study, no animals became ill or died prior to the experimental endpoint. All animals utilized in this study were euthanized by means of i.p. injection of pentobarbital after experiments. All animal experiments were conducted according to the national and international guidelines contained in the ‘Act on Welfare and Management of Animals’ (Ministry of Environment of Japan) and ‘Regulation of Laboratory Animals’ (Kyushu University) and under the protocols approved by the Institutional Animal Care and Use committee review panels at Kyushu University.

### Measurement of Ca^2+^ responses in 96-well plates (FDSS 7000EX system)

Measurement of Ca^2+^ imaging in 96-well plates was performed using the Functional Drug Screening System 7000EX (FDSS7000EX, Hamamatsu, Japan). 1321N1 cells stably expressing rat P2X4R and rat P2X7R were cultured for 24 h at 37°C on 96-well plates (2.0 × 10^4^ cells/well). Cells were loaded with 2.5 μM Fura-2-AM containing 0.02% pluronic (Molecular Probes) in a balanced salt solution (BSS; 150 mM NaCl, 5 mM KCl, 1.8 mM CaCl_2_, 1.2 mM MgCl_2_, 10 mM D-glucose and 25 mM HEPES, pH 7.4) at room temperature for 45 min and then washed with BSS. After pretreatment with the test drugs for 10 min, cells were stimulated with 10 μM ATP or 100 μM BzATP.

### Measurement of Ca^2+^ responses in single cells (single cells analysis)

Measurement of Ca^2+^ responses in single cells were assessed by ratiometric images (F340/F380) of Fura-2 fluorescence, which were detected with Aquacosmos/HiSca (Hamamatsu Photonics, Hamamatsu, Japan). 1321N1 cells stably expressing human P2X4R were transferred to poly-L-lysine-coated glass coverslips, placed in silicon rubber walls (Flexiperm, Greiner Bio-One GmbH, Frickenhausen, Germany) (1.0 × 10^4^ cells /well). 1321N1 cells were cultured for 24 h at 37°C and loaded with 2.5 μM Fura-2 -AM in BSS at room temperature for 45 min. Rat microglial cells were obtained as floating cells over the mixed glial culture. The floating cells were collected by gentle shaking and transferred to poly-L-lysine-coated glass coverslips, placed in Flexiperm (5.0 × 10^4^ cells/well). Microglial cells were cultured for 3–6 h and were loaded with 5 μM Fura-2-AM in DMEM at 37°C for 30 min. Then, the cells were washed with BSS and mounted on an inverted fluorescence microscope (ECLIPSE TE2000-U; Nikon, Tokyo, Japan) equipped with a Xenonlamp (Xe75W; Nikon). Data were calculated using the relative increase ratio (F340/F380) from the basal level prior to ATP application with or without duloxetine. The effects of duloxetine were evaluated by the S2 (2nd [Ca^2+^]_i_ response) / S1 (1st [Ca^2+^]_i_ response), where [Ca^2+^]_i_ represents intracellular Ca^2+^ concentration, or the ratio of the fluorescence intensity (F340/F380). Following addition of nucleotides (10, 50 or 100 μM ATP, 100 μM BzATP, or 10 μM ADP) for 30 s, the cells were pretreated with duloxetine for 10 min. The cells were pre-incubated with ivermectin (3 μM) for 3 min prior to ATP application.

### Intrathecal administration

Under isoflurane (2% [v/v]) anesthesia, a 32-gauge intrathecal catheter (ReCathCo, Allison Park, PA) for intrathecal administration of duloxetine (20 and 50 μg/20 μL) was inserted through the atlanto-occipital membrane into the lumbar enlargement and externalized through the skin according to a method described previously [5,16].

### Neuropathic pain model and behavioral test

We used the spinal nerve injury model [17] with some modifications. A unilateral L5 spinal nerve of rats was tightly ligated and cut just distal to the ligature under isoflurane (2.5%) anesthesia. To assess mechanical allodynia, calibrated von Frey filaments (0.4–15 g, Linton Instrumentation, Diss, Norfolk, UK) were applied to the plantar surface of the hindpaw from below the mesh floor. The 50% paw-withdrawal threshold (PWT) was determined by the up-down method [18]. Duloxetine (20 or 50 μg/20 μl) or vehicle (Phosphate buffered saline, 20 μl) was intrathecally administered to rats 7 days after PNI and mechanical allodynia was measured for 300 min. The paw withdrawal threshold at 180 min after intrathecal administration of duloxetine was converted into antiallodynic effect (%) which was calculated by the formula: antiallodynic effect (%) = 100 × (test value−control value)/(15 g−control value). Control and test values were paw withdrawal threshold at 0 and 180 min, respectively. PCPA (300 mg/kg, an inhibitor of 5-HT synthesis) was intraperitoneally administered once a day for 3 days from day 4 post-PNI, based on published studies [19]. DSP-4 (50 mg/kg, a NAergic neurotoxin) was intraperitoneally administered 3 days before PNI, based on published studies [20]. PCPA and DSP-4 were dissolved in saline.

### Statistical analysis

**Fig 1:**
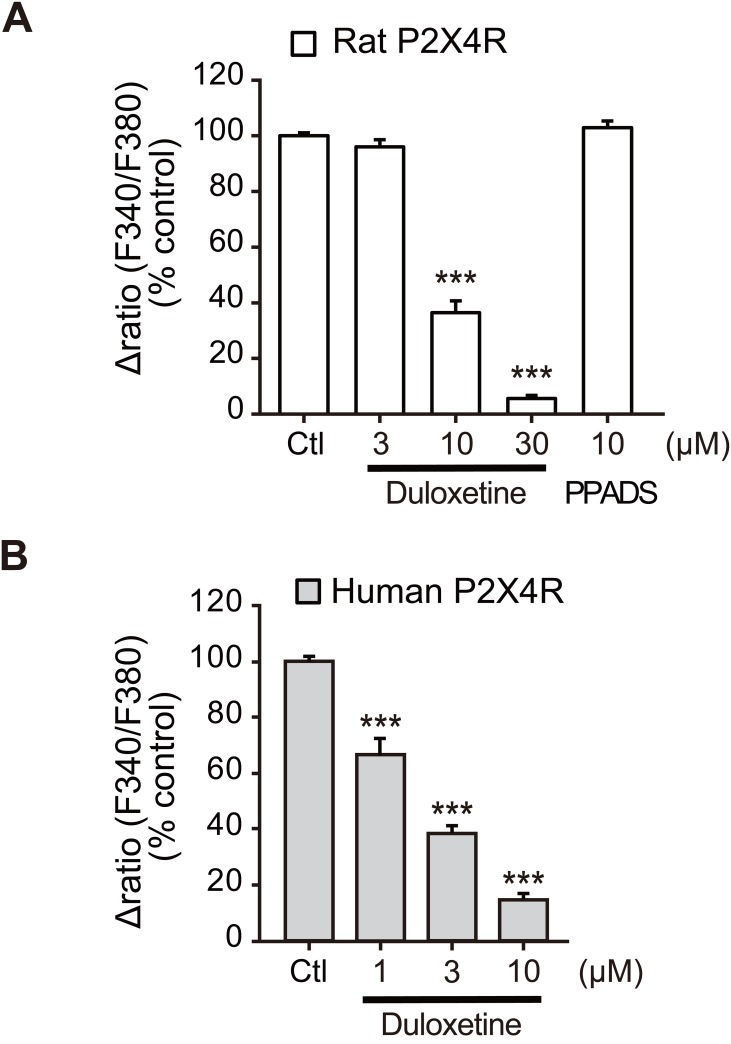
Duloxetine inhibits function of recombinant P2X4R. (A) Effects of duloxetine (3–30 μM) and PPADS (10 μM) on ATP (10 μM)-induced [Ca^2+^]_i_ responses in rP2X4R-1321N1 cells measured by the FDSS 7000EX system. Each column indicates the value as a percentage of control (Ctl: 10 μM ATP alone-induced [Ca^2+^]_i_ responses) (n = 4, ***P < 0.001 vs. control). (B) Effect of duloxetine at various concentrations (1–10 μM) on ATP (10 μM)-induced [Ca^2+^]_i_ respoznses in hP2X4R-1321N1 cells measured at a single cell level. Each column shows the effect of duloxetine evaluated by the S2 (2nd [Ca^2+^]_i_ response) / S1 (1st [Ca^2+^]_i_ response) (n = 4, ***P < 0.001 vs. control). Cells were pretreated with duloxetine and PPADS for 10 min prior to application of ATP. Data represent mean ± SEM for all groups.

**Fig 2:**
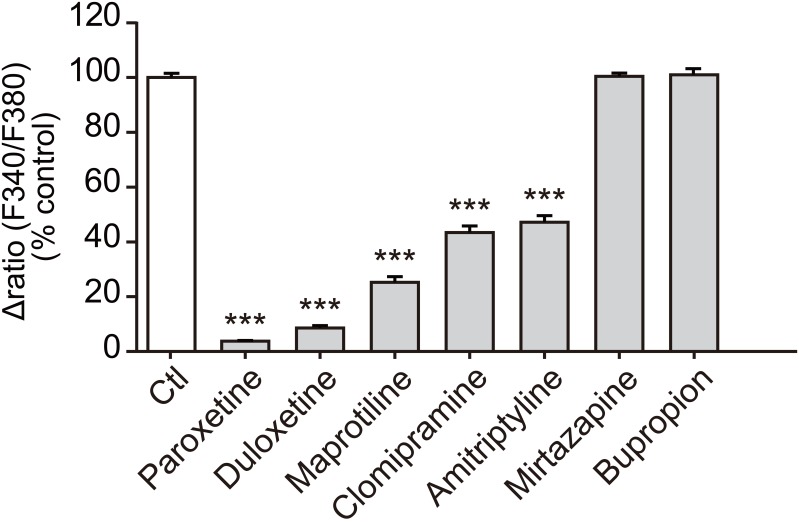
Comparison of the effect of duloxetine and other antidepressants on recombinant rP2X4R. Effect of duloxetine and other antidepressants (30 μM) on ATP (10 μM)-induced [Ca^2+^]_i_ in rP2X4R-1321N1 cells measured by the FDSS 7000EX system. Each column shows the value as a percentage of control (10 μM ATP alone-induced [Ca^2+^]_i_ responses) (n = 6, ***P<0.001 vs. control). Cells were pretreated with duloxetine and other antidepressants 10 min prior to ATP application. Data represent mean ± SEM.

**Fig 3:**
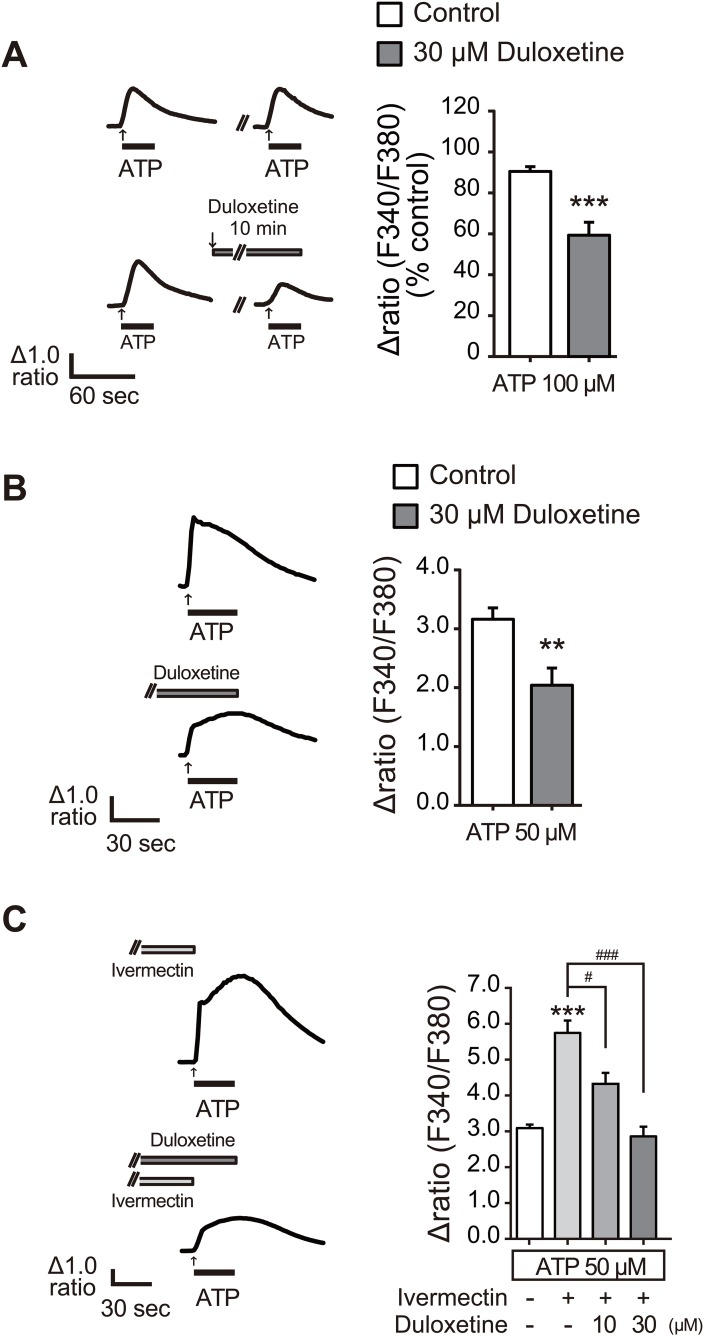
Duloxetine inhibited microglial P2X4R function. (A) Representative average traces of [Ca^2+^]_i_ increases in response to ATP (100 μM for 30 s) with or without duloxetine (30 μM for 10 min) measured by a single cell analysis in C8-B4 cells. Each column shows the effect of duloxetine evaluated by the S2 (2nd [Ca^2+^]_i_ response) / S1 (1st [Ca^2+^]_i_ response) (n = 4, ***P < 0.001 vs. control). (B) Representative average traces of [Ca^2+^]_i_ increases in response to ATP (50 μM for 30 s) with or without duloxetine (30 μM for 10 min) measured by single cell analysis in rat microglial cells. Each column shows the relative increase ratio (F340/F380) from the basal level prior to ATP application (n = 4, **P < 0.01 vs. control). (C) Representative average traces of [Ca^2+^]_i_ increases in response to ATP (50 μM for 30 s) in combination with ivermectin (3 μM, 3 min) with or without duloxetine (30 μM for 10 min) measured by single cell analysis in rat microglial cells. Each column shows the relative increase ratio (F340/F380) from the basal level prior to ATP application (n = 4, **P < 0.01, ***P < 0.001 vs. control, ^#^P < 0.05, ^###^P < 0.001). Data represent mean ± SEM for all groups.

**Fig 4:**
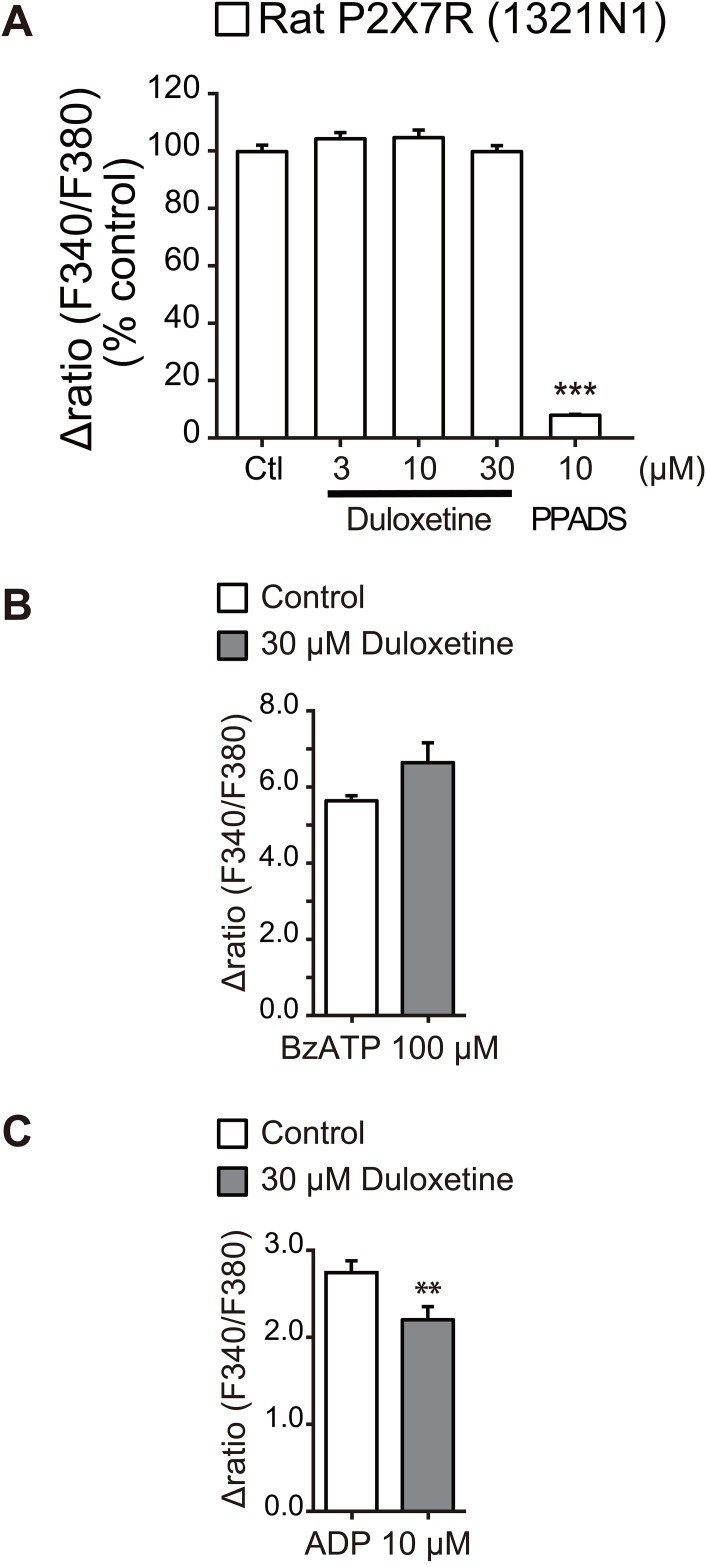
Effect of duloxetine on other P2 receptors. (A) Effect of duloxetine at various concentrations (3–30 μM) and PPADS (10 μM) on BzATP (100 μM)-induced [Ca^2+^]_i_ responses in rP2X7R-1321N1 cells measured by the FDSS 7000EX system. Each column shows the value as a percentage of control (100 μM BzATP alone-induced [Ca^2+^]_i_ responses) (n = 4). (B, C) Effects of duloxetine (30 μM) on [Ca^2+^]_i_ responses induced by BzATP (100 μM) (B) and ADP (10 μM) (C) measured by a single cell analysis in primary cultured rat microglial cells. Each column shows the relative increase ratio (F340/F380) from the basal level prior to BzATP or ADP application (n = 4, **P < 0.01). Cells were pretreated with duloxetine or PPADS for 10 min prior to application of BzATP or ADP. Data represent mean ± SEM for all groups.

**Fig 5:**
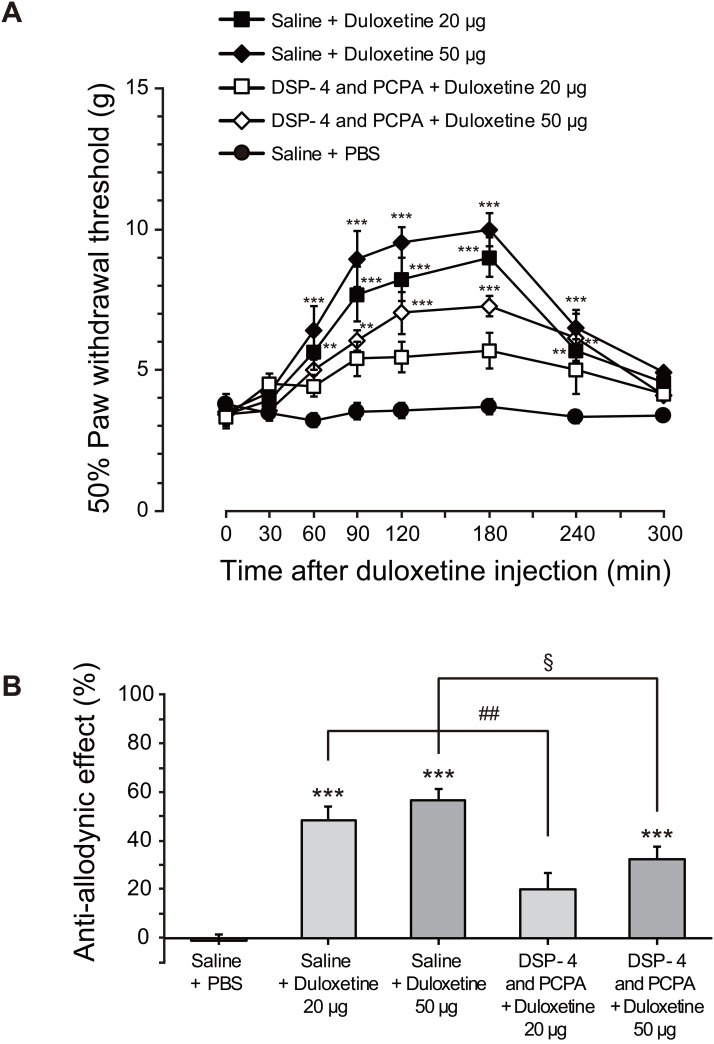
Intrathecal duloxetine attenuates allodynia after PNI: possible involvement of P2X4R. (A) The paw withdrawal threshold (grams) of mechanical stimulation by von Frey filaments applied to the rat hindpaw after PNI. Duloxetine (20 or 50 μg/20 μl) or vehicle (Phosphate buffered saline, 20 μl) was intrathecally administered to rats on day 7 post-PNI. PCPA (300 mg/kg, i.p.) was administered once a day for 3 days from day 4 post-PNI, and DSP-4 (50 mg/kg, i.p.) was administered 3 days before PNI. The threshold was measured 300 min after duloxetine injection (n = 5–13, **P < 0.01, ***P < 0.001). Data represent mean ± SEM. (B) The antiallodynic effect (%) of duloxetine at 180 min after its intrathecal administration was calculated by the formula: antiallodynic effect (%) = 100 × (test value−control value)/(15 g−control value) [***P < 0.001 vs control group (Saline+PBS), §P < 0.05 and ##P < 0.01]. Data represent mean ± SEM.

Statistical analyses were performed using one-way ANOVA with Bonferroni’s multiple comparison test (Figs 1A–1B and 2), Student’s t test (Fig 3A–3B), one-way ANOVA with Bonferroni’s multiple comparison test (Figs 3C and 4A), Student’s t test (Fig 4B–4C), two-way ANOVA with post hoc Bonferroni test (Fig 5A) or one-way ANOVA with post hoc Tukey Multiple Comparison test (Fig 5B) using GraphPad Prism 4 software. Differences were considered significant at P < 0.05.

## Results

### Duloxetine inhibits function of recombinant P2X4R expressed in 1321N1 cells

We screened 1979 compounds to identify clinically approved drugs that inhibit P2X4R. Using a high-throughput Ca^2+^ imaging apparatus, we measured [Ca^2+^]_i_ levels in 1321N1 human astrocytoma cells (1321N1 cells) stably expressing rP2X4R (rP2X4R-1321N1) [13]. We identified the SNRI duloxetine as a compound that has an inhibitory effect on ATP-induced increases in [Ca^2+^]_i_, and that is known to possess both oral availability and the ability to cross the blood-brain barrier. The inhibitory effect of duloxetine was concentration dependent in rP2X4R-1321N1 cells (Fig 1A). In Ca^2+^ imaging analysis at the single cell level using hP2X4R-1321N1 cells, duloxetine produced similar inhibitory effects, with an IC_50_ value of 1.59 μM (Fig 1B). These results indicate that duloxetine has an inhibitory effect on recombinant rat and human P2X4R.

We also tested two other antidepressants categorized into different classes that had not been previously tested, mirtazapine (NAergic and specific 5-HTergic antidepressant) and bupropion (NA-dopamine reuptake inhibitor). However, these two antidepressants at 30 μM had no effect on the ATP-evoked [Ca^2+^]_i_ responses in rP2X4R-1321N1 cells (Fig 2). In agreement with previous reports [13,21], paroxetine, maprotiline, clomipramine and amitriptyline significantly inhibited rat P2X4R-mediated [Ca^2+^]_i_ responses, and the inhibition by duloxetine was similar to that of paroxetine (Fig 2).

### Duloxetine inhibited microglial P2X4R function

In the central nervous system, microglia predominantly express P2X4R. Therefore, we examined the effect of duloxetine using C8-B4 cells, a cell line of immortalized mouse cerebellar microglial cells. We found that duloxetine reduced the ATP-induced [Ca^2+^]_i_ increase (Fig 3A). We then assessed its effect in rat primary cultured microglial cells that endogenously express P2X4R [4,15]. Duloxetine (30 μM) significantly reduced the ATP-evoked [Ca^2+^]_i_ responses in microglial cells (Fig 3B). To selectively detect a P2X4R-mediated component, we co-applied ATP with macrocyclic lactone antibiotic ivermectin, which is a positive allosteric modulator specific for P2X4R [22]. Consistent with previous studies, ivermectin enhanced the ATP-induced [Ca^2+^]_i_ responses (Fig 3C). This enhancement was almost completely suppressed by duloxetine (30 μM) (Fig 3C), suggesting an inhibitory effect of duloxetine on endogenous P2X4R.

In addition to P2X4R, microglia express P2X7 receptors [23]. However, duloxetine (30 μM) had no effect on rP2X7R expressed in 1321N1 cells (Fig 4A). In rat primary cultured microglia, duloxetine (30 μM) had no effect on [Ca^2+^]_i_ responses evoked by BzATP, an agonist for P2X7R (Fig 4B). Microglia also respond to ADP via P2Y12 receptors (P2Y12R), a subtype that is crucial for the motility of microglial processes [24]. ADP evoked [Ca^2+^]_i_ responses, which were slightly, but significantly, reduced by duloxetine (Fig 4C). Taken together, these results suggest that duloxetine likely has an effect that is more selective to P2X4R.

### Intrathecal administration of duloxetine attenuates mechanical allodynia after PNI: a possible involvement of P2X4R

Our previous studies have implicated microglial P2X4R in mechanical allodynia after PNI [4,6,7,9,25,26]; therefore, we next investigated whether duloxetine has an antiallodynic effect. After PNI, the paw withdrawal threshold to mechanical stimulation markedly decreased, implying the development of mechanical allodynia. After intrathecally administered duloxetine (20 or 50 μg) to rats on day 7 post-PNI, we found that the decreased paw withdrawal threshold was significantly reversed (Fig 5A): at 180 min, 48% (20 μg) and 57% (50 μg) recovery of paw withdrawal threshold (Fig 5B). Furthermore, we examined the antiallodynic effect of duloxetine in rats that had been pretreated with PCPA to deplete 5-HT, and with DSP-4 to produce a toxic effect on NAergic neurons that fully depletes 5-HT and NA [20,27,28,29]. In these PCPA- and DSP-4-pretreated rats, we found that the reversal effect of intrathecal duloxetine on the PNI-induced allodynia (Fig 5A): at 180 min after the injection of duloxetine, the pretreatment with PCPA and DSP-4 suppressed the duloxetine’s effect (P < 0.05 and 0.01; Fig 5B). However, the antiallodynic effect of duloxetine still remained at a statistically significant level (Fig 5A and 5B). These results suggest it is possible that an antiallodynic effect of duloxetine in our model of neuropathic pain may be associated, at least in part, with its inhibitory effect on P2X4R.

## Discussion

Our screening of clinically approved drugs revealed for the first time that the SNRI duloxetine has an inhibitory effect on P2X4R. We and others have previously reported that some antidepressants inhibit P2X4R-mediated Ca^2+^ responses [13,21,30]; in this study, the IC_50_ value of duloxetine for hP2X4R was 1.59 μM. This indicates duloxetine is as potent as paroxetine, which previously found to be the antidepressant with the most potent inhibition of P2X4R. Furthermore, at the concentration range eliciting P2X4R inhibition, duloxetine had no effect on P2X7R. This is in contrast to paroxetine which also inhibits P2X7R [13,31]. The tricyclic antidepressant amitriptyline has been reported to inhibit rat and mouse, but not human P2X4R [21]; in this study, the inhibitory effect of duloxetine was observed in rodent and human P2X4R, indicating the inhibition is not restricted to a specific species. Moreover, the P2X4R inhibition by duloxetine is not restricted to recombinant P2X4R. In our experiments, we showed the ability of duloxetine to inhibit P2X4R endogenously expressed in primary cultured microglial cells. It is well-known that microglia express several P2 receptors in addition to P2X4R [23], but by using ivermectin, a positive allosteric modulator specific for P2X4R [22], we showed a marked inhibition of P2X4R-mediated response by duloxetine. These results together indicate that duloxetine has an inhibitory effect on P2X4R expressed in microglial cells.

Studies have shown that duloxetine has attenuating effects on pain hypersensitivity in models of neuropathic pain associated with traumatic nerve injury [32,33,34,35], diabetic neuropathy [36,37] and fibromyalgia [38]. In addition to its clinical use for depression, duloxetine is frequently used for treating chronic pain such as diabetic neuropathic pain [39,40], chronic low back pain [41,42], osteoarthritis [41,43] and fibromyalgia [41,44,45]. It has been considered that the mechanism for pain relief by duloxetine might be distinct from its effect on depression [46]. The primary action of duloxetine is inhibition of 5-HT and NA reuptake into presynaptic terminals by their respective transporters. Thus, it is thought that the attenuating effects of duloxetine on neuropathic pain are related to an increase in the levels of 5-HT and NA in the descending pain inhibitory pathways from brainstem to the spinal dorsal horn [47,48]. Indeed, it has been reported that alleviation of mechanical allodynia in model of neuropathic pain by duloxetine was reduced by an intrathecal injection of a 5-HT_2A_ receptor antagonist [36,49] or by an intraperitoneally injection of DSP-4 to produce a toxic effect on NAergic neurons [20]. Consistent with these findings, the present study showed that the suppressing effect of duloxetine on PNI-induced allodynia was reduced in rats that had been pretreated with PCPA and DSP-4, at the dose of these agents needed to fully deplete 5-HT and NA [20,27]. However, the antiallodynic effect of intrathecal duloxetine still remained in the rats treated with both PCPA and DSP-4. Thus, it is possible that, in addition to the primary pharmacological action of duloxetine to inhibit 5-HT and NA transporters, an inhibitory effect on microglial P2X4R in the spinal cord may be involved in the antiallodynic effect of duloxetine when administered intrathecally in a model of neuropathic pain. In support of this idea, we previously demonstrated that expression of the P2X4R is upregulated exclusively in microglia in the spinal cord after PNI and that pharmacological blockade and molecular knockdown of the P2X4R produces a reversal of mechanical allodynia [4]. P2X4R-deficient mice also display a marked suppression of the PNI-induced mechanical allodynia [9,10]. Furthermore, intrathecal administration of paroxetine and fluvoxamine, which inhibits P2X4R, also produces a similar reversal of the PNI-induced allodynia, but citalopram, a SSRI that has no effect on P2X4R, does not suppress the allodynia [13]. Thus, these data support our hypothesis that an antiallodynic effect of duloxetine may be associated, at least in part, with its inhibitory effect on P2X4R.

In models of neuropathic pain, expression of P2X7R and P2Y12R are also increased in spinal microglia, and pharmacological blockade and genetic knockout of these receptors reduces pain hypersensitivities after PNI [50,51,52,53,54,55,56]. In this study, we observed that duloxetine had no effect on P2X7R, and produced a weak inhibition of the ADP-induced [Ca^2+^]_i_ responses in primary cultured microglial cells, which might be mediated by P2Y12R. Thus, it is possible that intrathecally administered duloxetine may slightly attenuate P2Y12R function in spinal microglia, which may also be involved, at least in part, in duloxetine’s effect on mechanical allodynia after PNI.

## Conclusions

In this study, we provide evidence for the first time that the SNRI duloxetine has an inhibitory effect on recombinant P2X4R (rat and human) and microglial P2X4R (mouse and rat). Furthermore, intrathecally administered duloxetine produced a reversal of mechanical allodynia, and the effect was reduced but still remained in rats pretreated with both a 5-HT depleting agent and a NAergic neurotoxin. Thus, we hypothesized that, in addition to the primary action of duloxetine on monoamine transporters, inhibition of P2X4R may be involved in duloxetine’s antiallodynic effect in a model of neuropathic pain.
